# Estimating effects of arable land use intensity on farmland birds using joint species modeling

**DOI:** 10.1002/eap.1875

**Published:** 2019-03-15

**Authors:** Martin Stjernman, Ullrika Sahlin, Ola Olsson, Henrik G. Smith

**Affiliations:** ^1^ Department of Biology, Biodiversity Unit Lund University Sölvegatan 37 Lund SE 223 62 Sweden; ^2^ Centre for Environmental and Climate Research Lund University Sölvegatan 37 Lund SE 223 62 Sweden

**Keywords:** agriculture, agri‐environment measures, farmland birds, intensification, Joint Species Distribution model, production land

## Abstract

Declines in European farmland birds over past decades have been attributed to the combined effects of agricultural intensification and abandonment. Consequently, aspirations to stop declines should focus attention on reversing these changes through voluntary or policy‐driven interventions. The design of such interventions should ideally be informed by scientific knowledge of which aspects of the transformation of agricultural landscapes have contributed to the farmland bird declines. Declines may be associated with loss of natural habitats or the intensification and homogenization of land use management on production land, and furthermore, these changes may interact. Here, we applied an orthogonal design exploiting spatial variation in land use in a major agricultural region of Sweden to seek evidence for benefits to farmland birds of reversing some of the intensifications on and among arable fields and whether effects are modified by the availability of seminatural habitats (pastures and field borders) in the landscape. We accounted for the potentially confounding effect of interactions between species by using a joint species distribution model explicitly controlling for additional variation and covariation among species. We found that interventions aimed specifically at land in production could provide benefits to farmland birds. Landscapes with a higher proportion leys or fallows and/or with a more diverse set of crops held higher abundances of most farmland birds. However, effects were only apparent in landscapes with low availability of seminatural habitats and were sometimes even negative in landscapes with high amounts of such habitats, demonstrating context dependence. Even if we found little evidence of interactions between species, the joint modeling approach provided several benefits. It allowed information to be shared between species making analyses robust to uncertainty due to low abundances and provided direct information about the mean and variability in effects of studied predictors among species. We also found that care needs to be taken regarding prior and distributional assumptions as the importance of species interactions might otherwise be overstated. We conclude that this approach is well suited for evaluating agricultural policies by providing evidence for or against certain interventions or to be linked to policy scenarios of land use change.

## Introduction

### Agricultural intensification and biodiversity

Decades of bird monitoring in Europe and North America have revealed marked declines of many farmland‐associated species (Fuller et al. 1995, Gregory and van Strien 2010, Reif 2013, Green and Lindström 2015). Concomitant changes in agricultural management and land use have led researchers to attribute these declines to the combined effects of agricultural abandonment and intensification (Chamberlain et al. 2000, Donald et al. 2001, Wretenberg et al. 2007, Stoate et al. 2009). Agricultural abandonment has been particularly prevalent in less fertile and/or remote regions, whereas agricultural intensification including landscape simplification and increased use of external inputs has characterized arable farming in core agricultural regions (Ihse 1995, Shrubb 2003). In livestock‐based systems intensification includes more intensive fodder production and less reliance on unimproved pasture (Vickery et al. 2001). To reverse the declines of birds in agricultural landscapes, e.g., through interventions such as agri‐environment schemes (AES) and cross‐compliance regulations under the Common Agricultural Policy (CAP), or through voluntary interventions, it is important to understand which aspects of the transformation of agricultural landscapes during the past decades that has contributed to the farmland bird declines (Newton 2004). This is inherently difficult, since agricultural intensification consists of a suite of inter‐correlated changes.

It has been suggested that a major reason for loss of farmland biodiversity is a loss of ecological heterogeneity at multiple spatial scales (Benton et al. 2003). At a large spatial scale, farmland birds may have suffered from regional farm specialization on either animal husbandry, with arable fields largely being used for fodder production, or crop farming without animal husbandry (Robinson et al. 2001, Atkinson et al. 2002, Benton et al. 2003). As a result, the farmland habitat on a scale relevant for birds has lost much of its heterogeneity and hence the ability to provide necessary and complementary resources for nesting and feeding (Benton et al. 2003, Vickery and Arlettaz 2012). At smaller spatial scales, the landscape has been simplified through the conversion of seminatural habitat, including permanent field borders, to arable fields or non‐agricultural habitat while variation between arable fields have been reduced by shorter crop rotations among fewer but more intensively grown crops (Atkinson et al. 2002, Stoate et al. 2009). Such simplified landscapes may not be able to provide resources for birds to nest and feed, because the lack of fields with different sowing times (autumn vs. spring) and/or structure (e.g., cereal grasses vs. broadleaved crops vs. seminatural grassland) results in farms not providing a suitable mix of dense (e.g., autumn‐sown cereals) and open (e.g., spring‐sown cereals and broadleaved crops) habitats needed by many species during the breeding season (Whittingham et al. 2006). A reduction in between‐field heterogeneity would also reduce the ability of the farmed landscape to harbor bird species with different feeding and nesting strategies (e.g., ground vs. foliage feeding or predator avoidance relying on crypsis vs. vigilance; Fahrig et al. 2011, Vickery and Arlettaz 2012). More complex crop rotations may increase heterogeneity by including multiyear leys and fallows that may allow the build‐up of insect and plant populations important as food resources for farmland birds (Barker et al. 1999, Bracken and Bolger 2006, Meiss et al. 2010). At the field level, ecological heterogeneity is lost through the increased use of fertilizers and pesticides, resulting in denser and more homogeneous crops with less weeds and grasslands with denser swards that are harvested more frequently (Chamberlain et al. 2000, Fuller 2000, Benton et al. 2003, Wilson et al. 2005). This may further reduce spatial complementarity between areas with dense swards and interspersed open spots within fields (Odderskær et al. 1997, McCracken and Tallowin 2004). Thus, given the importance of heterogeneity at the landscape to field scale for farmland bird biology, the spatial and temporal conformity induced at these scales by agricultural intensification is likely a major culprit in the demise of farmland bird biodiversity.

Because farmland bird declines are associated both to the loss of more natural (non‐crop) components of the farmed landscape, and to the intensification and homogenization of land use management on production land, policies to conserve farmland biodiversity should not only focus on preserving or reintroducing non‐crop habitat, but also on reversing aspects of intensification on production land (Fahrig et al. 2011, Lindsay et al. 2013, Santana et al. 2017). In light of the ongoing debate about whether conservation of farmland biodiversity should go via land sparing (taking land out of production to recover natural habitats) or land sharing (improving habitat quality on production land), the quantification of potential gains to biodiversity from alternative use of production land may be highly relevant (Green et al. 2005, Fischer et al. 2008). However, this requires knowledge about which heterogeneity matters.

### Gathering evidence

Ideally, the choice of measures to support by AES, include in cross‐compliance, or to propose as voluntary actions to farmers should be based on scientific evidence of what effects these interventions have (Dicks et al. 2013, 2014). However, experimental data on the effect of interventions on birds rarely exist, in particular for interventions at larger spatial scales such as multiscale heterogeneity (Fuller 2000). Furthermore, such evidence is often restricted to a few bird species, because of difficulties in estimating responses for more than the most common bird species or a single focal rare species. An alternative route to gather evidence is to capitalize on existing spatial and/or temporal variation in land use present throughout a region or time period of interest and design biodiversity surveys in which most of the variation along such gradients is captured (Gabriel et al. 2010, Fahrig et al. 2011). Although not experimental sensu stricto, survey designs in which orthogonality and coverage of relevant land use predictors are taken into account can be viewed as pseudo‐experimental and used to estimate the association between land use and presence and/or abundance of organisms (Pickett 1989, Fahrig et al. 2011). It may also be easier to disentangle the impact of different factors using a spatial approach, since simultaneous temporal changes in managements tend to result in a lack of orthogonality between factors (Chamberlain et al. 2000, Berg et al. 2015). However, evaluations based on space‐for‐time substitutions may not be able to focus directly on interventions per se, because they are too rare (e.g., skylark plots in Sweden) or too ubiquitous (e.g., effects of cross‐compliance system or “the greening of the CAP”) to be analyzed alone, but may rather reveal information about the importance of different *types of habitat and habitat heterogeneity* to birds, that in turn may be targeted by the interventions.

### Assessing impacts of land use on biodiversity

Model‐based approaches (sensu Warton et al. 2015b) such as linear models relating a response variable (e.g., species abundance) to a set of predictors (e.g., different land uses) can be used to inform decisions. Such approaches can readily handle (or propagate) sources of uncertainty and account for sources of variability in data (non‐normality, variance‐to‐mean relationships, overdispersion). To study effects of land use on bird communities, species’ abundance (or presence/absence) data can be modeled separately in single species models and combined into a summarizing community index afterward (e.g., the Farmland Bird Index; Gregory et al. 2005, Chiron et al. 2013, Stjernman et al. 2013). Separate species models may be of limited use for rare species where low counts and an excess of zeros yield parameter estimates with high uncertainty. Combining several independently run species models will also not take species interactions into account and results may therefore be biased (Baselga and Araújo 2010, Guisan and Rahbek 2011, Clark et al. 2014). An alternative approach is to a priori combine species in a common index, but this may mask contrasting effects of land use for individual species leading to dismissal of effects that could be highly influential for some of the included species. A third approach is to relate multiple response variables to predictors using “joint models” (also known as Joint Species Distribution models [JSDM]; Pollock et al. 2014).

In JSDMs, several response variables’ association to predictors is modeled simultaneously and thus not treated as independent (Ovaskainen et al. 2010, Clark et al. 2014, Pollock et al. 2014, Warton et al. 2015a). Instead, variance and covariance in and among species responses across sampled sites are explicitly modeled and hence accounted for. Variance and covariance among species can stem from direct biotic interactions between species (competition or facilitation), as well as from effects of unmeasured characteristics of the sites at which species are studied (Ovaskainen et al. 2017). Explicit modeling of this variance‐covariance both takes care of the potential noise and bias introduced by non‐independence in species responses (Clark et al. 2014) and handles species‐specific extra (residual) variation among sites (overdispersion). The estimates of the extra variance and covariance in species responses from external sources can be compared to effects of the included predictors and give information on the relative importance of each source of variation (Ovaskainen et al. 2017). Importantly, JSDMs make it possible to get parameter estimates for species whose rarity would not allow separate modeling, because effects of predictors are modeled hierarchically as coming from a (normal) distribution common to all included species while reducing the risk of over‐parameterization. Due to their complexity, joint models are often analyzed in a Bayesian statistical framework, which results in an output comprised of samples from the posterior distribution of parameters such that uncertainty around estimates can be easily and naturally propagated to the level of prediction if the model is to be used as such (Link and Barker 2010).

### Aim

Our aim is to use the joint modeling approach to seek evidence for the potential gain for farmland birds from alterations to the management of production land. We focus on alterations reversing some of the intensifications that has resulted in a reduction in habitat heterogeneity, particularly on and among arable fields: (1) a *simplified crop rotation* in which periods of extensive cropping (leys or fallow) has been largely excluded, (2) *reduced crop diversity* focusing on a limited set of profitable crops (mainly cereals and oil‐seed rape), and (3) an increased *dominance of autumn‐sown crops*.

Our sampling design exploits existing spatial variation in agricultural land use in a major agricultural region in southern Sweden and care is taken to produce a data set where orthogonality between land uses is preserved as much as possible. The benefit of habitat heterogeneity induced by more diverse cropping patterns on arable fields may depend on the availability of alternative heterogeneity‐inducing structures in the landscape such as permanent seminatural pastures and the non‐crop habitats of field borders (Tscharntke et al. 2005, Kleijn et al. 2011). Hence, the variance in land use is set against a gradient in landscape heterogeneity as determined by these two variables, likewise acknowledging the goal of orthogonality.

We apply the joint modeling approach within a Bayesian framework and investigate the model's sensitivity to prior and distributional assumptions. Our approach yields posterior distributions of parameters that can easily be applied to future scenarios for land use and thus to inform decisions about policies such as future CAP reforms.

## Materials and Methods

### Study region and design

**Figure 1:**
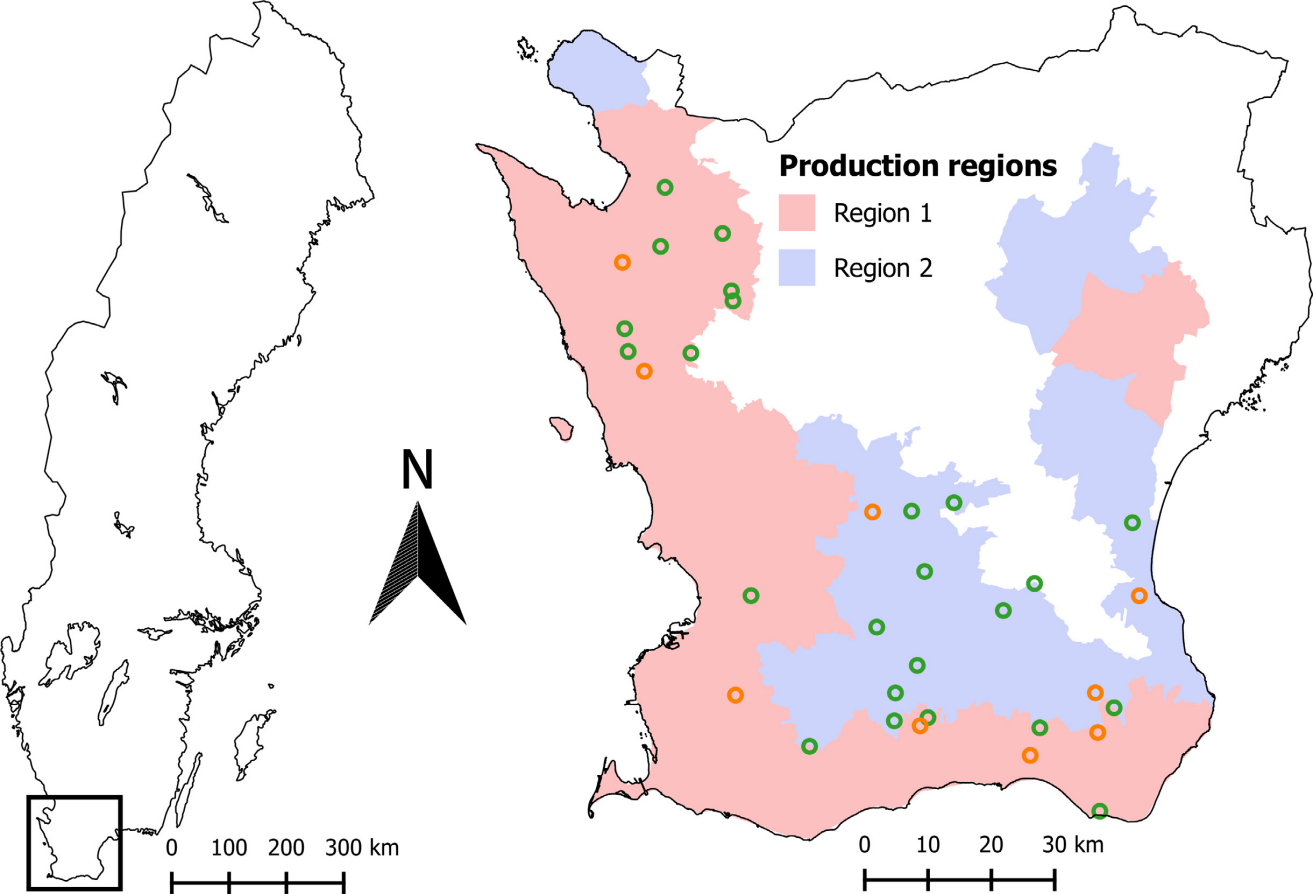
Map of the geographical distribution of study landscapes in Scania, southern Sweden. Ley design landscapes are shown in green and flower strip landscapes in orange. The delineation of production region 1 (Open plains) and 2 (Mixed farmland) in Scania is also shown.

The southwestern and central parts of Scania (the southernmost province of Sweden; Fig. 1) are dominated by farmland and characterized by a large variation in both agricultural productivity and landscape heterogeneity, ranging from intensive farming with large annually tilled fields to mixed farming with smaller fields and a larger proportion of leys and seminatural grazed pastures (Persson et al. 2010). While at the regional scale, this gradient has a (southwest [intensive]‐northeast [extensive]) geographical orientation, this is less apparent at more local scales (a few kilometers). Hence, this region is well suited to produce a study design that optimizes orthogonality between variation in aspects of agricultural intensification and a background of varying landscape heterogeneity, i.e., where these variables vary more or less independently.

We took advantage of data from a project designed to study effects of various management alternatives under varying landscape heterogeneity (Rusch et al. 2013, Williams and Hedlund 2013, Andersson et al. 2014, Rader et al. 2014). In particular, the data we used in this study come from two designs of the project studying the importance of having ley (Andersson et al. 2014) or flower strips (Jönsson et al. 2015), respectively, in the landscape. In this project, study landscapes were placed in the most productive regions of Scania in such a manner as to maximize orthogonality between landscape heterogeneity and the set of landscape predictors studied. Hence, the two study designs produced a data set maximizing orthogonality between ley or flower strips and landscape heterogeneity. Given the structure of the Scanian agricultural landscape, these designs as a side effect resulted in a combined data set also having low collinearity between many other landscape characteristics, which was exploited in the present study (we did not explicitly evaluate the original designs here). Here we describe in detail the design approach taken in the ley design part, but the technique was the same for the flower strip design. Predictor information was extracted from the ARARAT database, an integrated administration and control system (IACS) database maintained by the Swedish Board of Agriculture for administrating CAP's area‐based subsidies. This database contains information on agricultural land use (area and types of crops grown) in spatially explicit land use parcels (blocks).

Potential study landscapes (sites) were 1 km radius circular areas centered at individual farms in the most to medium productive regions of Scania (production regions 1 and 2, respectively; see Fig. 1). The farm‐centered approach was necessary to accommodate requirements of the original studies. Since our focus was farmland, we included only landscapes within these regions with more than 40% farmland. The gradient in landscape heterogeneity was captured by combining the proportion of seminatural pasture and field borders in the landscape. In Scania, these habitats are strongly correlated and describe well the transition from large arable fields with few (semi)natural habitats to smaller arable fields interspersed with more non‐crop farmland (including seminatural pastures). It might be argued against describing landscape heterogeneity by means of the amount of seminatural pastures and field borders in the landscape, as maximum heterogeneity would rather be attained at intermediate levels of such seminatural habitats and a landscape composed of 100% of these land uses may be viewed as homogeneous at the other side of the spectrum. However, in this agricultural region and at the scale at which this study was conducted (1 km radius landscapes), the percentage of the agricultural land covered by permanent seminatural pastures and field borders exceeds 50% in only a very few extreme areas (two military training areas), the vast majority (>90%) having <20% seminatural cover. Further, the permanent seminatural pastures are generally composed of a more heterogeneous set of (micro‐)habitats (bushes, trees, small wetlands, etc.) compared to arable fields. Under these circumstances, we conclude that it is valid to base the measure of landscape heterogeneity on these two variables. It also brings the benefit of utilizing the same measure of landscape heterogeneity as in previous studies exploiting the same study design (Rusch et al. 2013, Williams and Hedlund 2013, Andersson et al. 2014, Rader et al. 2014).

The proportion of seminatural pasture and field border (assuming a field border breadth of 1 m effectively turns field border length into an area) of total farmland area in the landscape was combined by extracting the first principal component (PC1) from a principal component analysis (PCA) of the two variables. Proportion seminatural pasture was square‐root transformed to improve linearity between the variables (i.e., pasture was transformed to a scale comparable with field border, which is in practice a length). We also calculated the proportion of ley in the landscape. Ley is part of the crop rotation in these regions and will hence vary in amount between years; we therefore used the average proportion of ley in the three previous years to estimate the expected amount of ley in the landscape the coming year. Proportion ley was calculated relative to the total amount of farmland in the landscape.

An orthogonal design (i.e., producing a data set in which the correlation between ley and landscape heterogeneity is minimized) was then established by plotting average proportion of ley against landscape heterogeneity for all farm‐centered landscapes in the region and selecting study landscapes representing as much as possible of the two‐dimensional space (Fig. 1).

The farm‐centered approach used when designing the study means that the sample of landscapes on which we performed the PCA is partly biased toward a higher representation of farms from the more heterogeneous region (where farms are smaller and more aggregated). We therefore calculated a more representative measure of landscape heterogeneity for the whole region by performing a PCA using the same two variables as above (standardized to a mean of zero and standard deviation of one), but on a set of non‐overlapping 1‐km landscapes covering approximately 80% of production region 1 and 2 in Scania. Non‐overlap and maximum coverage was ensured by placing a hexagonal grid of landscapes within the two regions centered such that as many landscapes as possible were fitted within region borders. We then calculated landscape heterogeneity index (LHI) scores for our study landscapes on the scale of the first principal component (PC1) from the PCA of these representative (non‐overlapping) landscapes using the means (indicated by overbars) and standard deviation (SD) of the respective variables from the representative landscape set

$$\text{LHI}=\frac{\sqrt{2}}{2}\left(\frac{{\sqrt{\text{prop.pasture}}}_{\mathrm{study}}-\bar{\sqrt{{\text{prop.pasture}}_{\mathrm{representative}}}}}{\text{SD}\left(\sqrt{{\text{prop.pasture}}_{\mathrm{representative}}}\right)}+\frac{\text{prop.field}\;{\text{border}}_{\mathrm{study}}-\bar{\text{prop.field}\;{\text{border}}_{\mathrm{representative}}}}{\text{SD}\left(\text{prop.field}\;{\text{border}}_{\mathrm{representative}}\right)}\right).$$

The original PC1 score based on farm‐centered landscapes at the design stage and the calculated LHI score based on the representative landscapes were highly correlated (*r*_Pearson_ > 0.99), and only the latter was used in further analyses.

### Land use variables measuring agricultural intensification

The choice of input variables describing various aspects of the intensification of agriculture that has occurred in recent decades is based on the set of available land uses in the IACS database. The IACS database provides the most detailed and spatially explicit data on land use available for the agricultural landscape in Sweden. Since it is designed for administration of subsidies for farmers rather than to describe the landscape as habitat for birds, we had to combine land use categories into new variables to capture different aspects of intensification of functional importance to birds.

#### Proportion extensive cropping (PE)

This variable intends to measure the role of temporally intermixing intensive crop production (annual crops) with more extensive land use (ley or fallows) in the rotation. In relation to intensive cropping, leys and fallows are both expected to result in less intense land use in terms of levels of inputs and frequency of disturbance. It was calculated as the proportion of the summed area of ley and fallow on total arable (tillable) land area (i.e., excluding permanent seminatural grasslands) in the landscape.

#### Extensive quality (EQ)

From a bird's perspective, leys and fallow may be quite different land uses. Fallows are likely to provide a better habitat for many farmland birds than rotational leys. In fact, even leys can be of different quality; long‐laying or extensively managed leys should exhibit a more diverse grass flora and heterogeneous structure and as such are more similar to fallow than are short rotation leys. The IACS database provides two different codes for ley where one is assigned to managed leys with a specified set of cultivated grass species while the other is used for leys that do not conform to the subsidy rules of containing only cultivated grasses. Often the latter class consists of leys that have not been managed (cultivated and reseeded) for some time and on which a larger variety of grass and weed species have invaded and where the sward is more heterogeneous. This class of ley may hence be more similar to long‐term fallow in terms of bird habitat quality. To reflect this distinction in habitat quality of managed leys vs. “non‐managed” leys and fallows we included the proportion of the sum of the latter two among all extensive land use [(fallow + non‐managed ley)/(fallow + all ley)]. This predictor was square‐root transformed to ensure an even distribution among study landscapes.

#### Crop diversity (CD)

This variable measures the role of maintaining a high diversity of crop types between agricultural fields in the landscape and is calculated as the Shannon diversity (Jost 2006) of crop types. Crops from the IACS database (excluding pasture) were grouped into 13 structural crop types based on structural as well as temporal characteristics of the crops assumed to be relevant to birds (Josefsson et al. 2017). For example, spring‐sown cereals constitute one group temporally separate from autumn‐sown cereals, which in turn are structurally separate from, e.g., spring‐ and autumn‐sown oil‐seed rape, respectively (Appendix S1 Table S1 for a detailed description of the grouping applied). The area of each of these 13 structural crop types in the landscape was used in the calculation of Shannon diversity.

#### Proportion spring sown crops (PSS)

One aspect of the intensification of agriculture is the transition from spring‐sown to autumn‐sown crops. Spring‐ and autumn‐sown crops differ in several ways as habitat for farmland birds. Timing of cultivation, sward development, and harvest, as well as the presence of over‐winter stubble are all characteristics that differ between the two sowing regimes and should potentially affect birds. We calculated this as the proportion of cereals and oil‐seed rape that were spring sown.

#### Other inputs

We also included farmland area (FA) and LHI as predictors. Farmland area is included to cover the fact that survey landscapes, although having more than 40% farmland, may still differ considerably in amount of farmland. Given the strong connection to farmland of the studied species, this difference between landscapes introduces extra noise, which needs to be controlled for. Furthermore, including farmland area will provide information about the importance of agricultural abandonment on bird biodiversity. As already described, we wanted to study effects of land use against a background of landscape heterogeneity, and hence, LHI is included as both a main effect and in interactions with the land use intensity variables. The main effect of LHI is of interest in itself as it is a composite of seminatural pastures and field borders in the landscape and as such can be viewed as a proxy for the amount of (semi)natural non‐cropped land adding to the habitat mosaic of the agricultural landscape (Vickery and Arlettaz 2012). Such habitats provide the main nesting and feeding resources to some of the farmland bird species and serve as complementary habitats for others.

### Bird surveys

**Figure 2:**
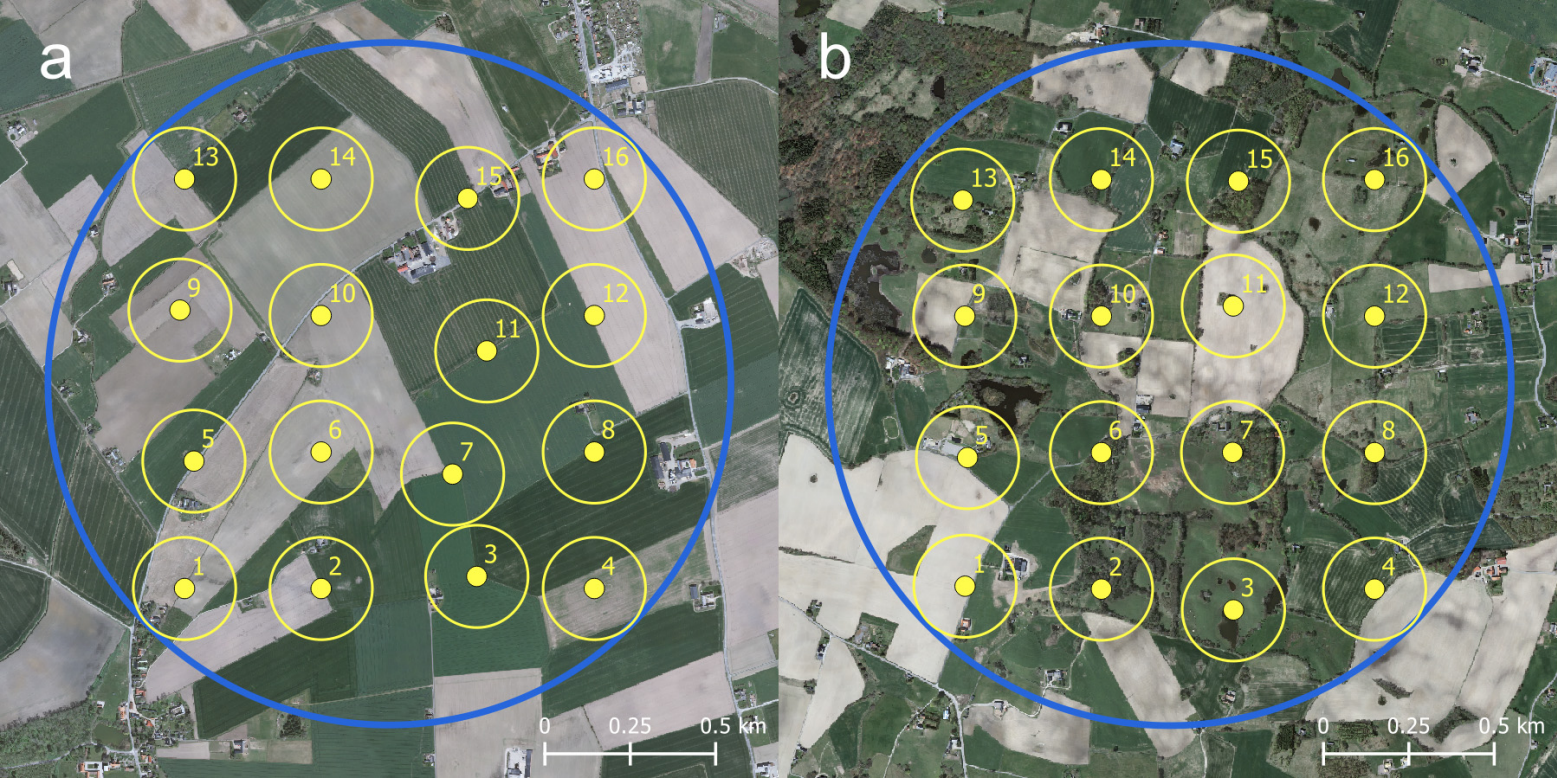
Examples of (a) homogeneous and (b) heterogeneous landscapes (radius 1 km) in the data set. Survey points are depicted in yellow as are circles showing maximum distance (150 m) at which bird observations were included in analyses. Background map: GSD‐Ortofoto, 1‐m resolution, © Lantmäteriet (2012; The Swedish National Land Survey).

Within each landscape, 16 survey points were placed in a regular grid (4 by 4; 400 m in between) numbered from lower left moving first eastward and then northward (Fig. 2). Odd numbered points were moved to the nearest field border to accommodate needs of other studies using this design. Field border is defined as the border of the IACS land parcels (blocks) and can border both farmland and non‐farmland. Points ending up far outside agricultural land (i.e., more than 50 m into forests, urban areas or water) were excluded, and hence, the number of points surveyed varies somewhat between landscapes (range 11–16 points).

Landscape visits were divided among five different observers making sure that no landscape was visited twice by the same observer and that the timing of visits was not related to the characteristics of the landscapes. Each survey point was visited twice during the breeding season 2011 (mid May–mid June). The species and abundance of all bird observations, by sight or hearing, during a 5‐minute period were recorded on orthophoto maps (covering an area of approximately 500 × 500 m; scale 1:2,500). Birds too far away or out of sight such that the observer was unable to accurately specify the location on the map were excluded. Flying birds that landed or took off during the observation were noted as observed at the point of landing or take‐off, while continuously flying birds were noted at the point of the observed flight trajectory closest to the observer. The bird observations on the orthophoto maps were later digitized (into a point‐geometry shape‐file) using ArcGIS (ESRI 2010) and transferred into program (R Development Core Team 2017) for further handling.

The spatial specificity of the observations enabled calculations of the distance at which birds were seen. Based on expert opinion on reasonable observation distances for most species, we decided to only include observations made within 150 m from the observation point. For each of the two visits and for each species, observations of individual birds were summed at landscape level. We also kept track of the number of points surveyed at each visit in each landscape to be able to control for this in the analyses.

Counts of Starlings (*Sturnus vulgaris*) required a special treatment. This species exhibit synchronized fledging within a few days in late May–early June. The fledging date of the study region in 2011 was easily discernable in the survey data where Starling numbers rose sharply after 4 June. After this date, observations of Starlings consisted largely of flocks containing both adults and young birds. As our focus here was adult birds, we discounted Starling observations for the influx of young birds by dividing counts made after 4 June by 3, hence assuming the proportion of adult birds in Starling flocks was 1/3. This proportion was based on data from a Swedish environmental monitoring program under the Swedish Environmental Protection Agency in which the average number of fledged young per pair for a nest‐box population in the Scanian region was estimated at 4.01 for the years 2001–2009 (Svensson 2010).

### Farmland birds

Our definition of farmland birds in this study is based on their inclusion in two biodiversity indicators annually reported to the Swedish Environmental Protection Agency as part of the regular biodiversity monitoring program of Sweden (see Table 1; Green and Lindström 2015). The Swedish Farmland Bird Index (FBI) is used by Swedish authorities as a general indicator of how biodiversity fares within Swedish agriculture and consists of 14 of the farmland bird species included in the European counterpart that regularly occur in Sweden (data *available online*).^1^ The other indicator is coupled to one of Sweden's 16 environmental objectives (objective no 13 “A varied agricultural landscape”) and is composed of a set of 13 farmland bird species, largely overlapping with the FBI. We combined the two groups of species yielding a set of 15 species for use in the modeling (two species in each indicator are unique, and one of these does not occur as a regular breeder in the study region).

**Table 1:** Names of the species included in the analysis, the indicator to which they belong, and their mean abundance across landscapes (maximum over visits)

Species	Latin name	Indicator	Mean abundance
Barn Swallow	*Hirundo rustica*	FBI, EO	8.8
Curlew	*Numenius arquata*	EO	0.3
Greater Whitethroat	*Sylvia communis*	FBI, EO	10.6
Lapwing	*Vanellus vanellus*	FBI, EO	3.9
Linnet	*Carduelis cannabina*	FBI, EO	4.7
Meadow Pipit	*Anthus pratensis*	FBI	0.5
Red‐backed Shrike	*Lanius collurio*	FBI, EO	0.2
Rook	*Corvus frugilegus*	FBI	14.4
Skylark	*Alauda arvensis*	FBI, EO	24.9
Starling	*Sturnus vulgaris*	FBI, EO	10.7
Tree Sparrow	*Passer montanus*	FBI, EO	16.4
Wheatear	*Oenanthe oenanthe*	EO	0.8
Whinchat	*Saxicola rubetra*	FBI, EO	1.0
Yellow Wagtail	*Motacilla flava*	FBI, EO	2.2
Yellowhammer	*Emberiza citrinella*	FBI, EO	4.1

*Note*

FBI, Farmland Bird Index; EO, Swedish Environmental Objective 13 “A varied agricultural landscape.”

### Statistical model

The joint species modeling followed the approaches of Ovaskainen et al. (2010) and Pollock et al. (2014) in which the association between a set of species and the environment is modeled by a multivariate hierarchical generalized linear model. These studies modeled species (co‐)occurrence (presence/absence) patterns, however, as our data consists of counts, we modified the model accordingly:

$$X_{i}B=\beta_{0}+\beta_{1}{\text{FA}}_{i}+\beta_{2}{\text{LHI}}_{i}+\beta_{3}{\text{PE}}_{i}+\beta_{4}{\text{EQ}}_{i}+\beta_{5}{\text{CD}}_{i}+\beta_{6}{\text{PSS}}_{i}+\beta_{7}{\text{LHI}}_{i}\times {\text{PE}}_{i}+\beta_{8}{\text{LHI}}_{i}\times {\text{EQ}}_{i}+\beta_{9}{\text{LHI}}_{i}\times {\text{CD}}_{i}+\beta_{10}{\text{LHI}}_{i}\times {\text{PSS}}_{i}$$

$$M_{i}∼N^{J}\left(X_{i}B,\Sigma \right)$$

$$Y_{\mathrm{ij}}=F\left(e^{M_{\mathrm{ij}}+{\mathrm{site}}_{i}+{\mathrm{observer}}_{o(i)}+\log_{e}{\mathrm{npoints}}_{i}},\theta_{j}\right)$$

$$B_{\mathrm{kj}}∼N\left(\mu_{k},\sigma_{B(k)}^{2}\right)$$

$${\text{site}}_{j}∼N\left(0,\sigma_{S}^{2}\right)$$

$${\text{observer}}_{o}∼N\left(0,\sigma_{O}^{2}\right).$$

The *J* species’ predicted abundances at site *i*,** M**_*i*_, was modeled on the log‐link scale as following a *J*‐dimensional multivariate normal distribution (*N* ^*J*^) with the *J* means linearly dependent on the predictors in the *i*th row of the design matrix **X** through a set of *K* parameters **Β** (*K* × *J* matrix) and a *J* × *J* variance‐covariance matrix **Σ**. Site (landscape) and observer random effects were added to the linear predictor to handle the fact that sites were visited twice and that there were five different observers. We also included an offset corresponding to the actual number (log‐transformed) of points surveyed at each site. The number of observed individuals of species *j* at site *i* (*Y*_*ij*_) was seen as drawn from a discrete distribution *F*(mean, dispersion) with mean set by the linear predictor on the data scale (*e* ^linear predictor^) and a species specific dispersion parameter θ_*j*_. We tried two discrete distributions commonly used to model counts: the Poisson distribution with dispersion θ_*j*_ = 1 for all *j* (i.e., variance = mean), and the negative binomial distribution where the dispersion parameter enters in the parameterization of the variance as μ* + *μ^*2*^ */*θ_*j*_. Species specific coefficients (Β_*kj*_) in the linear predictor were drawn from a normal distribution with hyper‐parameters μ_*B(k)*_ (mean) and σ^*2*^_*B(k)*_ (variance). This treatment of the land use effects as coming from a distribution common to all species enabled inferences to be made about the average effect among species of a specific land use (*k*) from the mean hyper‐parameter μ_*B(k)*_ as well as about the variance in effects of that land use among species from the variance hyper‐parameter σ^*2*^_*B(k)*_.

### Priors

A full account of priors for all parameters can be found in Table 2. As priors for variance (hyper) parameters (standard deviations; σ_*B(k)*_, σ_*S*_, σ_*O*_), we used the Cauchy^+^ (positive half‐Cauchy) distribution, and as priors for the mean hyper‐parameters (μ_*B(k)*_), we used the normal distribution. As parameters for these priors in turn, we set location (mean) to zero while we tried two alternative levels of regularization (i.e., “informativeness” or scale; see Table 2). Regarding the species specific dispersion parameter (θ_*j*_) of the negative binomial distribution, we used an exponential distribution as prior for its inverse (1/θ_*j*_) and likewise tried two levels of regularization by altering the rate parameter of the exponential distribution (λ = 0.5 or λ = 1). A higher λ value shrinks the exponential distribution toward zero and hence constrains the amount of extra variance (overdispersion or aggregation) under the negative binomial model.

**Table 2:** Prior specifications for parameters

Parameter	Prior
μ_*B(k)*_	**Normal(0, 100)**/Normal(0, 10)
*σ*_*B(k)*_	**Cauchy** ^**+**^ **(0, 5)**/Cauchy^+^(0, 2.5)
1/θ_*j*_	Exponential(0.5)/**Exponential(1)**
**Σ**	IW: **Σ** ~ inverseWishart(df, **I** ^*J*^)
	SIW: **Q** ~ inverseWishart(df, **I** ^*J*^)
	ξ_*j*_ ~ Normal^+^(0, 1)
	**Δ** = diagonal(**ξ**)
	**Σ** = **ΔQΔ**
	SSIW: **Q** ~ inverseWishart(df, **I** ^*J*^)
	**Δ**_*Q*_ = diagonal(1/√*Q*_jj_)
	**R** = **Δ**_*Q*_ **QΔ**_*Q*_
	σ_*j*_ ~ Cauchy^+^(0, 5)/Cauchy^+^(0, 2.5)
	**Δ** = diagonal(***σ***)
	**Σ** = **ΔRΔ**
	**SSLKJ**:** R** ~ LKJ(1)
	σ_*j*_ ~ **Cauchy** ^**+**^ **(0, 5)**/Cauchy^+^(0, 2.5)
	**Δ** = diagonal(***σ***)
	**Σ** = **ΔRΔ**
σ_*S*_	Cauchy^+^(0, 2.5)
σ_*O*_	Cauchy^+^(0, 2.5)

*Notes*

df = J + 1, **I** ^*J*^ = *J* by J identity matrix. Priors in boldface type resulted in the lowest widely available information criterion (WAIC) and were used when making inferences on parameters. *J* is the number of species.

The inverse‐Wishart distribution is commonly used as a prior for the variance‐covariance matrix **Σ** (Ovaskainen et al. 2010, Pollock et al. 2014). However, the inverse‐Wishart prior suffers from dependence between the on‐ and off‐diagonal elements of the variance‐covariance matrix (strong covariances are associated with large variances/standard deviations; Alvarez et al. 2014, Liu et al. 2016). Therefore, in addition to an inverse‐Wishart (IW) prior on **Σ**, we also tried three alternative priors that have been suggested to decouple this association. In the first of these, the scaled inverse‐Wishart prior (SIW), an inverse‐Wishart prior was set on an unscaled variance‐covariance matrix **Q**, which was then scaled by a vector of scaling parameters, given a normal^+^ (positive half‐normal) prior, to produce **Σ** (Gelman and Hill 2007). In the remaining two, a so called separation strategy (SS) was used where the variance‐covariance matrix was decomposed into a vector of standard deviations and a correlation matrix (Barnard et al. 2000, Alvarez et al. 2014, Liu et al. 2016). In one (SSIW), an inverse‐Wishart prior was set on a variance‐covariance matrix **Q**, which was then transformed into a correlation matrix **R** while in the other (SSLKJ) an LKJ prior (Alvarez et al. 2014, Stan Development Team 2016) was set directly on the correlation matrix **R**. We used Cauchy^+^ priors for the vector of standard deviations in both of these, similarly trying two levels of regularization (see Table 2).

### Implementation

Bayesian analysis was done by MCMC sampling using Stan via R (package rstan; R Development Core Team 2017, Stan Development Team 2017). We ran five chains with 4,000 iterations each of which one‐half were warm‐up yielding a total posterior sample of 10,000 iterations. The success of sampling from the posterior was determined based on inspection of parameter trace plots as well as on diagnostics output from Stan ($\hat{R}$, divergence warnings, E‐BFMI, etc.). Initial problems with the sampling were remedied by following the recommendations about vectorization and reparameterization in the Stan manual (Stan Development Team 2016). Relative model performance was determined by its out‐of‐sample predictive ability based on estimates of Widely Applicable Information Criterion (WAIC; McElreath 2016).

### Post‐posterior analysis

Acknowledging the Bayesian approach, we view the unknown parameters as laden with uncertainty and base inference on the samples from their posterior distributions (Link and Barker 2010, McElreath 2016). We summarized posterior samples as density plots, posterior means or medians (for variance parameters) with 95% credible intervals (CI; presented in the form mean_post_/median_post_ [CI]), and as specific probabilities (defined as the probability of the parameter being positive, *p*_positive_). The credible intervals are defined as the 95% probability interval with the highest posterior density (HPD). This was done on the log‐link scale in density plots and as the percentage change in species abundance, e.g., $100*\left(e^{B_{\mathrm{kj}}}-1\right)$, elsewhere. To illustrate effects of land use predictors involved in interaction with landscape heterogeneity, we present effects by combining the main and interaction effects, setting LHI at either its minimum or its maximum value, i.e., effects are presented for the least and most heterogeneous landscapes in our data set, respectively.

## Results

### Data summaries

**Figure 3:**
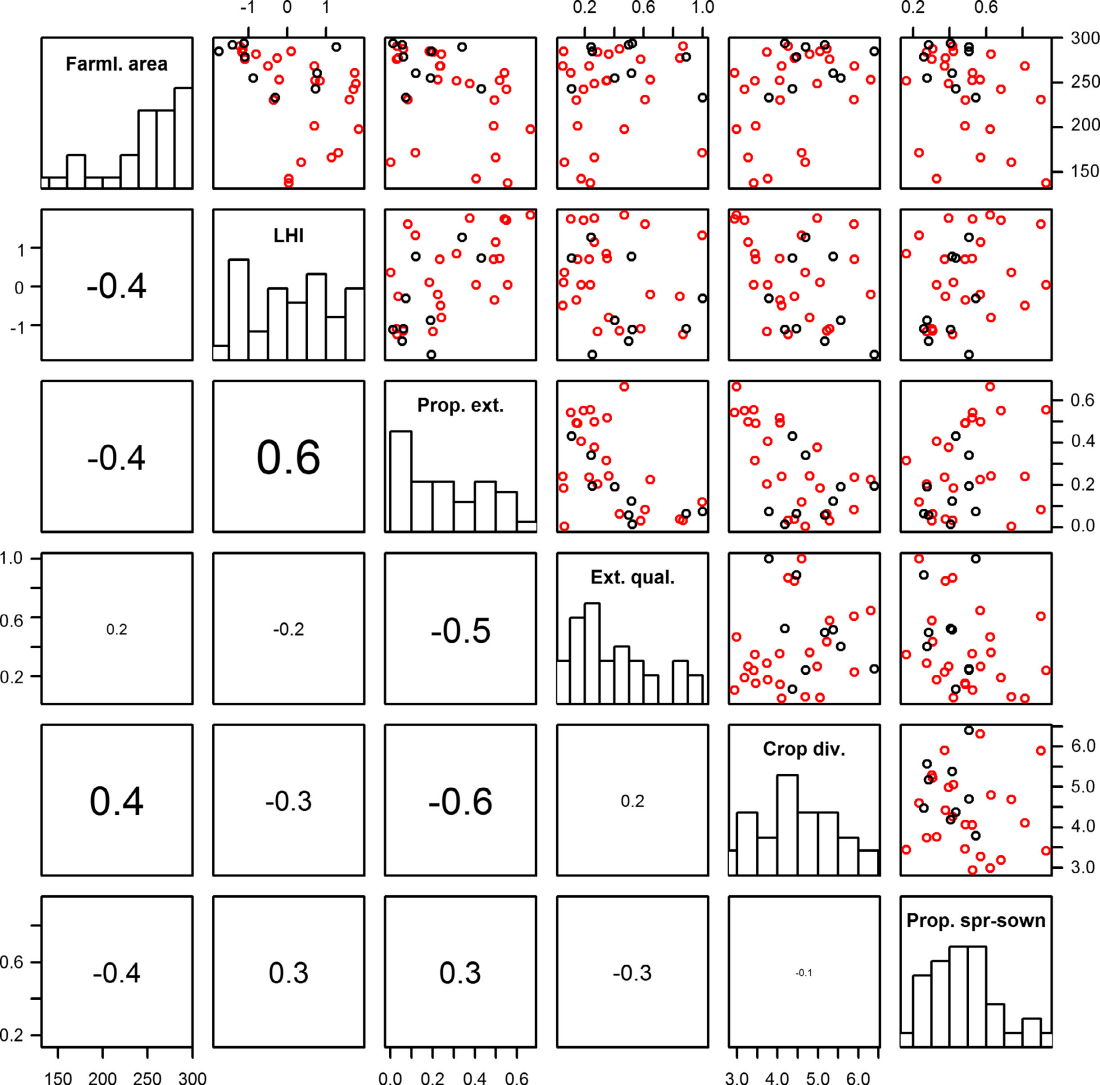
Relationships among the land use input variables modeled. Above diagonal plots are pairwise scatterplots of land use variables from the two designs (ley in red, flower in black). Below diagonal plots are the estimated correlation with size corresponding to absolute strength. Farml. area, Farmland area; LHI, Landscape heterogeneity index; Prop. ext., Proportion extensive cropping; Ext. qual., Extensive quality; Crop. div., Crop diversity; Prop. spr‐sown, Proportion spring sown crops

Taking the maximum over the two visits in each landscape, the total number of birds observed within 150 m within each point was 7,495 individuals of 97 species. Among the 15 farmland bird species, we observed a total maximum of 3,415 individuals (Table 1). Each species were on average observed in 22.9 (range = 5–33) landscapes. There was considerable variation in land use (Table 3) with low to moderate inter‐correlations such that variance inflation was consistently low (all below 3; Fig. 3).

**Table 3:** Summary statistics of the land use predictors included in the analysis

Input variable	Mean	SD	Range	Variance inflation factor
Farmland area	245.3	45.37	137.70–293.86	1.49
Landscape heterogeneity index	0.124	1.075	−1.762–1.843	1.67
Proportion extensive	0.260	0.196	0.00268–0.664	2.97
Crop diversity	4.481	0.939	2.938–6.398	1.68
Extensive quality	0.399	0.275	0.0495–1.000	1.58
Proportion spring sown	0.476	0.186	0.164–0.929	1.25

*Note*

Variance inflation was calculated using the corvif‐function from Zuur et al. (2013).

### Distributional and prior choices

All else equal, the negative binomial model clearly outperformed the Poisson model in terms of WAIC, with an average difference (∆WAIC) of 236 ± 66 (estimate ± SE). We also found that the negative binomial model with a more informative rate = 1 in the exponential prior for the inverse of θ performed better (∆WAIC ≥ 10 ± 2). The performance of the alternatively (more or less) informative hyper‐priors for land use dependencies (μ_*B(k)*_ and σ_*B(k)*_) was indistinguishable (∆WAIC ≤ 2 ± 1).

We found some differences in predictive ability between models with different priors for **Σ**. The IW prior performed worst and SSLKJ prior performed best (∆WAIC ≈ 60 ± 10). When using the IW prior, samples from the posterior distributions for some species showed dependence between σ_*j*_ and correlation in that higher σ_*j*_ was associated with stronger correlations (generally this was the case for correlations involving rarer species). This dependence was alleviated when using any of the alternative priors, especially so for priors using the separation strategy (SSIW, SSLKJ). We also found that a less informative prior on σ_*j*_ under separation strategy had lower WAIC than the more informative version.

### Correlation and variance in abundance

**Figure 4:**
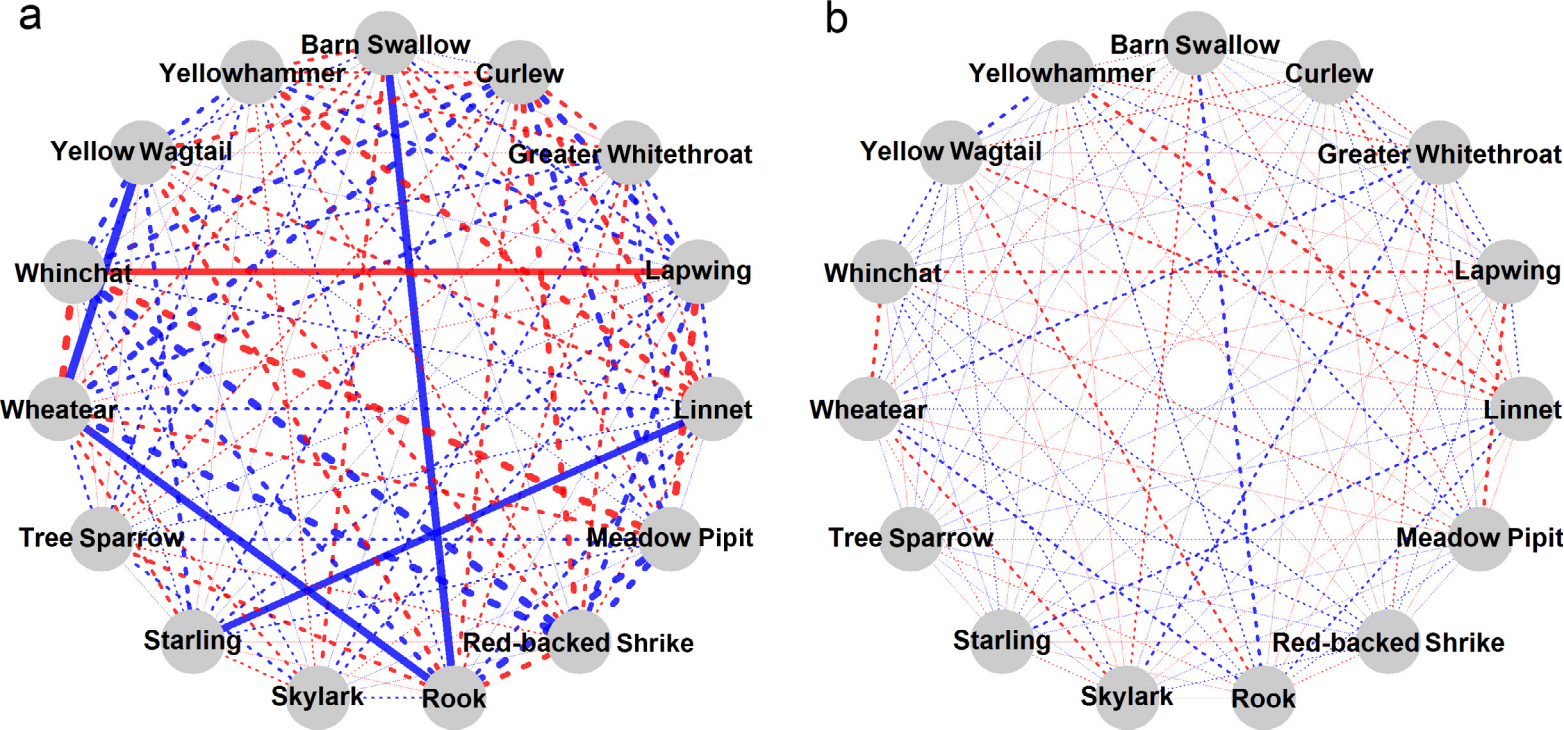
Comparison of residual correlations between species as estimated from (a) the Poisson model under an inverse‐Wishart (IW) prior and (b) the negative binomial model under a Lewandowski, Kurowicka, Joe (LKJ) prior. Pairwise correlations between species are depicted by lines where negative correlations are in blue and positive correlations are in red. Thickness of lines corresponds to the estimated correlation and solid lines are correlations where CI exclude zero and hatched lines where CI include zero.

We found very little evidence for residual correlations between species. Under the best performing model (negative binomial model with LKJ prior), there was no evidence for between‐site residual correlations between any of the species (strongest correlation ρ_Linnet – Yellowhammer_ = 0.25 [−0.14–0.61]) and uncertainty was very large (width of CI varied from 0.73 to 0.98). In general, the IW prior yielded on average twice as large (≈90–120%) estimates compared to the LKJ prior but they were also ≈30% more uncertain (width of CI varied from 0.90 to 1.46). Similarly, the Poisson model generally generated ≈30% stronger absolute estimates compared to a corresponding negative binomial model but, in contrast, these were often less uncertain (minimum CI using Poisson distribution = 0.63, Fig. 4). Thus, in the most extreme case (Poisson model with IW prior), there were four species pairs exhibiting correlations with credible intervals outside zero (e.g., ρ_Barn Swallow–Rook_ = −0.54 [−0.84 to −0.21], ρ_Lapwing–Whinchat_ = 0.47 [0.01–0.87]); however, the WAIC of this model was considerably higher than the best model (∆WAIC = 301 ± 64).

While we found no evidence of among site correlation, several species showed evidence of residual among site variance (here reported as standard deviations, σ_*j*_). However, the uncertainty in estimates was often substantial, especially (and expectedly) for rare species. Translated into percentages, examples of species with low residual variance were Tree Sparrow and Greater Whitethroat (σ_Tree Sparrow_ = 20% [0–63%], σ_Greater Whitethroat_ = 28% [0–51%]). Skylark and Linnet exhibited intermediate residual variance (σ_Skylark_ = 46% [12–84%], σ_Linnet_ = 97% [22–189%]) and Yellow Wagtail and Rook had high values (σ_Yellow Wagtail_ = 352% [70–940%], σ_Rook_ = 305% [76–665%]). The Wheatear was most extreme, both in terms of median and CI (σ_Wheatear_ = 815% [8–4715%]).

### Effects of land use

**Figure 5:**
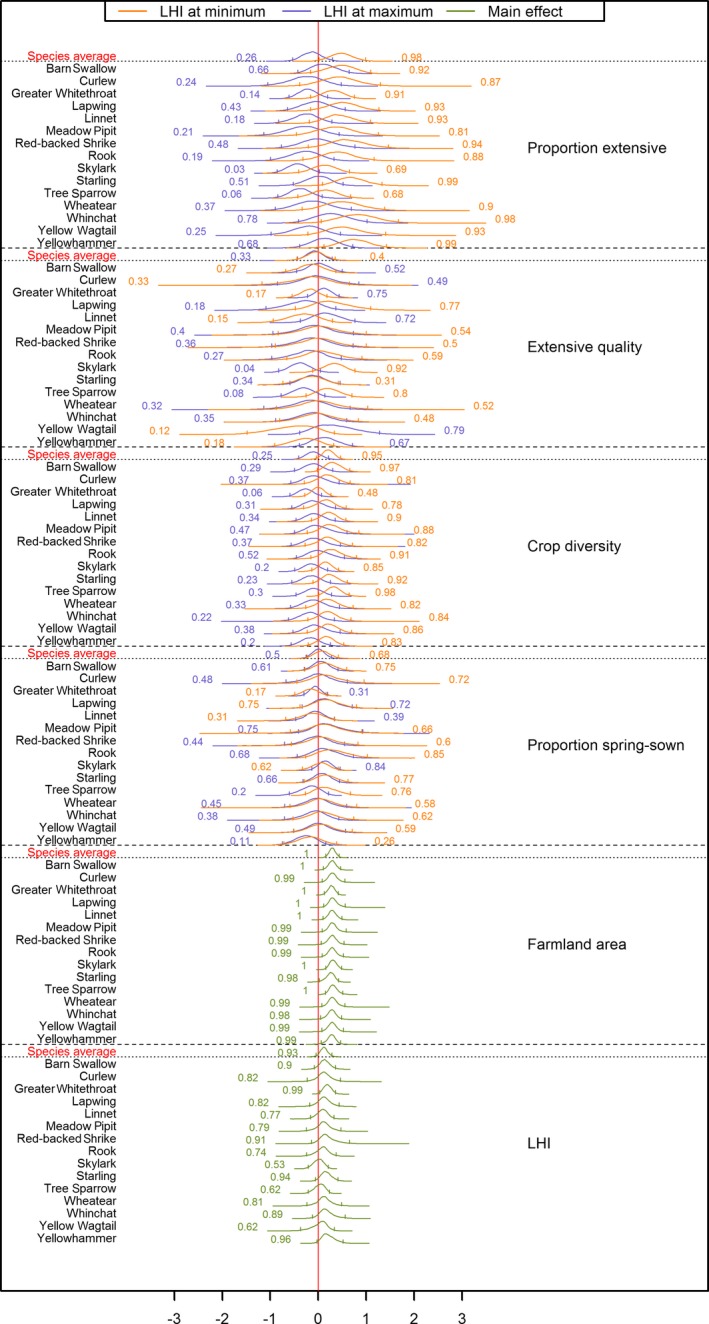
Density plots of 10,000 samples from the posterior distributions of the effect (on the log‐scale) of land use on species’ abundances. Two density curves on the same row describe the posterior distribution of the effect of the corresponding land use predictor when the landscape heterogeneity index (LHI) is held at its minimum (most homogeneous; orange‐red) or maximum (most heterogeneous; blue) value from the data set. Single density curves (green) describe effect distributions when other predictors are held at their mean. CI are shown by vertical bars. Numbers refer to the proportion of the posterior sample in corresponding color having values above zero (*p*_positive_).

As expected, farmland birds responded clearly and positively to the area of farmland (FA) in the landscape (Fig. 5). The species average effect from a 1 standard deviation (45 ha) increase in area of farmland (μ_FA_) was a 34% [15–53%] increase in abundance. The variation in this effect between species was low (σ_FA_ = 6% [0–21%]), ranging from *B*_FA_ = 28% [2–53%] for Starling to *B*_FA_ = 38% [10–73%] for Lapwing.

The average effect of landscape heterogeneity (LHI) was positive (μ_LHI_ *= *13% [−5–32%], *p*_positive_ = 0.93) and variation between species was moderate (σ_LHI_ = 14% [0–37%]). Five species had *p*_positive_ > 0.90 and all species had *p*_positive_ ≥ 0.53. Species associated with seminatural pastures or field borders showed more positive effects (e.g., Greater Whitethroat, Yellowhammer, Whinchat, Red‐backed Shrike; *B*_LHI_ ≥ 21%), while typical open field species showed no effects (e.g., Skylark and Yellow Wagtail; *B*_LHI_ = 1–4%).

Farmland birds were on average positively affected by the proportion of arable fields with leys or fallows (PE) in the most homogeneous landscapes (μ_PE:Low LHI_ = 67% [−7–149%], *p*_positive_ = 0.98) but not in the most heterogeneous ones (μ_PE:High LHI_ = −11% [−45–29%], *p*_positive_ = 0.26). This was also the land use predictor varying most in main effect between species (σ_PE:Main effect_ = 34% [9–70%]), while variation in interaction with LHI was low (σ_PE:Interaction_ = 9% [0–30%]). In the most homogeneous landscape, the specific probability for a positive effect was high (*p*_positive_ ≥ 0.9) for a majority of species (10 species) while none showed any evidence for a negative effect (*p*_positive_ ≥ 0.68). Notably, three species showed strong positive effects (Yellowhammer, *B*_PE:Low LHI_ = 125% [−2–282%], *p*_positive_ = 0.99; Starling, *B*_PE:Low LHI_ = 112% [−4–255%], *p*_positive_ = 0.99; Whinchat, *B*_PE:Low LHI_ = 152% [−17–372%], *p*_positive_ = 0.98). In the most heterogeneous landscape, two species showed clear evidence of being negatively affected by proportion of extensive cropping (Skylark, *B*_PE:High LHI_ = −33% [−60 to −3%], *p*_positive_ = 0.03; Tree Sparrow, *B*_PE:High LHI_ = −29% [−59–5%], *p*_positive_ = 0.06), while the rest was largely indifferent to this land use (*p*_positive_ = 0.14–0.78).

There was much less evidence that the quality of extensive cropping (EQ) influenced farmland birds. The average effect was low and similar along the landscape heterogeneity gradient (μ_EQ:Low LHI_ = −3% [−39–37%], *p*_positive_ = 0.40; μ_EQ:High LHI_ = −6% [−37–31%], *p*_positive_ = 0.33). Species varied little in main effect (σ_EQ:Main effect_ = 7% [0–23%]), while between species variation in the interaction with LHI was comparatively large (*σ*_EQ:Interaction_ = 25% [0–55%]). Exceptions to the general pattern were Skylark and Tree Sparrow, where especially the Skylark responded positively in the most homogenous landscape (*B*_EQ:Low LHI_ = 43% [−15–108%], *p*_positive_ = 0.92) but negatively in the most heterogeneous landscape (*B*_EQ:High LHI_ = −30% [−58–1%], *p*_positive_ = 0.04).

The response to crop diversity (CD) in the landscape was rather similar among species (σ_CD:Main effect_ = 15% [0–35%]; σ_CD:Interaction_ = 5% [0–18%]), but varied along the landscape heterogeneity gradient. There was a positive response in the most homogeneous landscapes (μ_CD:Low LHI_ = 24% [−6–56%], *p*_positive_ = 0.95), while there was basically no response in the most heterogeneous landscape (μ_CD:High LHI_ = −9% [−36–21%], *p*_positive_ = 0.25). Five species showed strong positive responses to crop diversity in the most homogeneous landscape (Tree Sparrow, *B*_CD:Low LHI_ = 40% [−1–88%], *p*_positive_ = 0.98; Barn Swallow, *B*_CD:Low LHI_ = 39% [−6–87%], *p*_positive_ = 0.97; Rook, *B*_CD:Low LHI_ = 38% [−20–112%], *p*_positive_ = 0.91; Starling, *B*_CD:Low LHI_ = 30% [−15–78%], *p*_positive_ = 0.92; and Linnet, *B*_CD:Low LHI_ = 27% [−16–75%], *p*_positive_ = 0.90). The Greater Whitethroat stands out as having the opposite pattern among the farmland birds, responding negatively to crop diversity in the most heterogeneous landscape (*B*_CD:High LHI_ = −22% [−46–5%], *p*_positive_ = 0.06) while not showing any response in homogeneous landscapes (*B*_CD:Low LHI_ = 0% [−25–29%], *p*_positive_ = 0.48).

We found no evidence that the proportion of spring‐sown (PSS) crops was an important predictor for farmland bird abundance and the effect was not influenced by landscape heterogeneity (μ_PSS:Low LHI_ = 10% [−24–47%], *p*_positive_ = 0.68; μ_PSS:High LHI_ = 1% [−24–29%], *p*_positive_ = 0.50). There was some indication of variation in main effects between species (σ_PSS:Main effect_ = 22% [0–50%]), but not in the interaction with landscape heterogeneity (σ_PSS:Interaction_ = 10% [0–27%]). Strongest positive effects were shown by Rook and Skylark in homogeneous and heterogeneous landscapes, respectively (Rook, *B*_PSS:Low LHI_ = 47% [−33–154%], *p*_positive_ = 0.85; Skylark, *B*_PSS:High LHI_ = 17% [−16–53%], *p*_positive_ = 0.84) while strongest negative effect was shown by Yellowhammer in heterogeneous landscapes (*B*_PSS:High LHI_ = −22% [−55–14%], *p*_positive_ = 0.11).

We chose only to report results from analyses of the full model since our aim was to evaluate the evidence for the full set of predictors. However, a few alternative reduced models were tested yielding very similar results and with similar predictive abilities. Due to space limitations, we do not present a full account of this process here, but lower WAIC was achieved using a reduced model without the predictors extensive quality (EQ) and proportion spring‐sown (PSS). However, out‐of‐sample predictive ability was not considerably improved compared to the full model (∆WAIC = 7 ± 7) and estimates of effects of remaining land use predictors were very similar although slightly strengthened (estimates approximately 8% stronger and uncertainty reduced by 2%; Appendix S1 Fig. S1).

## Discussion

In this study, we searched for evidence of what benefits could accrue to farmland birds from actions directed at the production component of the agricultural landscape (Fahrig et al. 2011, Tscharntke et al. 2012). We found that lowering land use intensity through the use of leys or fallows in the rotation and/or increasing habitat heterogeneity on arable fields by using a structurally more diverse set of crops could influence farmland bird abundance. Effects were dependent on the presence of seminatural habitats in the form of field borders and seminatural pastures in the landscape. In homogeneous landscapes (with few such habitats), estimated effects were largely positive and comparable to or even exceeding the role played by the area of farmland per se. However, even if there was a high probability that these actions would generate positive responses for most farmland birds in homogeneous landscapes, the uncertainty in estimates of effects was quite substantial and the possibility of weak or even negative effects cannot be dismissed for many species. Furthermore, the effects found in homogeneous landscapes contrast to those found in heterogeneous landscapes where effects were generally either weak or sometimes even negative.

### Landscape heterogeneity moderated effects

Our results conform to recent hypotheses that effects of land use management alternatives depend on the landscape context. Hypotheses differ as to the expectations of whether effects should be strongest in relatively simple (Tscharntke et al. 2005) or complex (Kleijn and Sutherland 2003) landscapes. This is generally believed to depend on the ecological contrast induced by the altered management, and where on the gradient of simplification (agricultural intensification or homogenization), the contrasted landscapes are positioned (Kleijn et al. 2011). While conservation actions are not believed to exert any effects in landscapes that are almost completely cleared of natural habitats and biodiversity (Tscharntke et al. 2005), they should have larger potential in simple (i.e., intensified but not completely cleared) compared to complex landscapes with lower intensity and where a large proportion of natural habitats is maintained (Batáry et al. 2011, Kleijn et al. 2011). Our results therefore suggest that, from the perspective of farmland birds, agricultural landscapes in the most productive part of Sweden range from simple (but not cleared) to complex in terms of agricultural intensification (Rundlöf and Smith 2006). This is because it was only in the most homogenous landscapes with a shortage of more natural habitats that actions reducing land use intensity on production land provided a large enough ecological contrast to show discernible effects.

### Benefits provided by extensified rotation

Seemingly, the strongest contrast was achieved by keeping a higher proportion of the arable fields as leys or fallows. This may be due to a higher amount of insect and weed resources compared to intensively cropped fields where these are controlled by higher levels of inputs (pesticides in particular) and cultivation. Furthermore, many farms including ley in rotation are also livestock farms and the effects we found may thus be correlated effects of animal husbandry (Musitelli et al. 2016). Particularly strong effects exhibited by typical grassland species such as Starling, Whinchat, and Yellowhammer suggest that cultivated grasslands per se provide important complementary or supplementary resources (Hiron et al. 2013). However, there was no evidence that the quality of the habitat (here measured as the proportion of these fields being long‐laying ley and fallows) mattered to almost any of the species. This was surprising as we had anticipated that the heterogeneous sward and/or richer weed flora characteristic of low (no) management habitats such as fallows and long‐laying leys would be preferred over the more intensive management on rotational leys with a higher amount of inputs (fertilizers) and more frequent mowing (Vickery et al. 2001). Low counts for some species could have limited the power to detect any extra benefit of fallows or long‐laying leys where it would have been expected (e.g., Whinchat, Meadow Pipit, Curlew). This contention is supported by the fact that a common species such as the Skylark (and to some extent Tree Sparrow) showed evidence of a positive response to fallows and long‐laying leys while being indifferent to rotational leys. However, it is less likely to explain the pattern exhibited by other rather common species.

We might also have underestimated the effects of ley or fallow quality. We have used the distinct IACS classification of leys in “approved” and “non‐approved” for ley subsidies to represent rotational and long‐laying leys, respectively, assuming that leys classified as “non‐approved” are fields taken out of crop rotation for long periods (no cultivation or reseeding), allowing a more diverse and heterogeneous flora to invade. Most often this is probably the case, however, there is the possibility that some of these grasslands may be disqualified for other reasons, for example, they may have temporarily been set aside for other uses (harvest storage, flooding, etc.) and, therefore, this class may exhibit quite large variation in quality for birds. Similar quality variation may also characterize fallows as their management prescriptions are not based on their value for biodiversity, such that prescriptions, e.g., allow them to be kept as bare soil and under repeated harrowing and herbicide treatment. In a similar vein, the large uncertainty that in general was found for effects of proportion of extensive cropping (PE) could be due to quality differences between leys used for silage (higher fertilizer input and more frequent mowing) vs. hay, neither of which could be discerned from IACS.

In accordance with theory (Kleijn et al. 2011), a high proportion of extensive cropping appeared not to constitute a significant contrast in heterogeneous landscapes for most species. For some species (e.g., Skylark and Tree Sparrow), the results even suggest that a high proportion extensive cropping may be negative. Despite our efforts toward an orthogonal study design, in those heterogeneous landscapes where extensive cropping dominates, it is largely composed of more intensively managed leys rather than long‐laying leys and fallows (Fig. 3), and furthermore, by necessity fields with annual crops (e.g., cereals) are rare. In comparison to managed leys, annual crops have been shown to hold higher densities of the field nesting Skylark (Donald and Vickery 2000) and similarly, arable fields with cereal or root crops have been shown to be especially important in regions dominated by grassland (Robinson et al. 2001). Hence, the shortage of annual crops and the dominance of intensively managed grasslands may explain the negative response to extensive cropping found in the most heterogeneous landscapes.

### Benefits from crop composition

A high diversity of crops with different structure and seasonality is assumed to enable more farmland birds to find both spatially and temporally complementary resources for feeding and nesting within their home range (Vickery and Arlettaz 2012) and hence may compensate for simplified landscape heterogeneity. Accordingly, several studies find that crop diversity enhance bird diversity (Firbank et al. 2008, Lindsay et al. 2013, Josefsson et al. 2017) but there are also examples of studies that have failed to find effects (Fahrig et al. 2015, Hiron et al. 2015). Our results partly corroborated the positive findings and especially, similar to Josefsson et al. (2017), we found that effects was limited to homogeneous landscapes where availability of interstitial seminatural habitats is low. In contrast, however, while these authors only found effects on non‐crop nesters, we did not see any evidence for differential effects related to nesting or other life‐history strategies. Rather the pattern was consistent across species such that although effects for individual species were low to moderate, the effect became clear when considering all species together.

While the structural appearance of different crop seemed to influence farmland bird abundance we found no evidence of a difference in effects between spring‐ and autumn‐sown crops. Autumn‐sown crops has been considered to be of lower quality for farmland bird (Newton 2004). For example, the development of the sward may be too advanced during the breeding season preventing access to food and nesting resources, the timing and amount of pesticide application differ and autumn‐sown crops are not associated with over‐winter stubbles (Donald and Vickery 2000, Newton 2004). However, some aspects of autumn‐sown crops suggest they may play an important role complementing spring‐sown crops. The denser sward may be richer in invertebrates and provide concealment from predators and therefore preferred by foliage‐gleaners. Furthermore, some species change preference over the season, starting off in autumn‐sown crop but later switching to spring‐sown (Eggers et al. 2011). Birds may therefore be dependent on both types of crop and an intermediate combination may be optimal. The benefit of a mixture of autumn‐ and spring‐sown crops might be captured by the crop diversity predictor where spring‐ and autumn‐sown cereals and oil‐seed rape constitute four different structural crop types and may be responsible for much of the variation in this predictor. Our study may also not capture the full benefits of spring‐sown crops. In particular, the over‐winter stubble often preceding spring‐sown crops is considered important to overwintering birds (Bradbury and Stoate 2000, Donald and Vickery 2000, Baker et al. 2012). These birds generally range over larger areas in winter and the positive effects might be diluted and not discernible in the local breeding populations that we are studying here. Furthermore, the majority of farmland birds studied do not winter in the local area from which our measure of spring‐sown crops originate.

### Among species variation in land use effects

The joint modeling provides estimates of the variation in effects of land use between species (σ_*B(k)*_). This information may be important for at least two reasons. First, knowledge about the extent of variability in effects of a certain intervention for different species is valuable when deciding among alternative actions. We found seemingly larger variability in effects of the proportion leys and fallows than in effects of crop diversity. The reasons for the difference in variabilities are unclear. It might be due to ecological differences between species, but it might also be due to our ability to correctly describe the two predictors based on the information in IACS. We have already described some problems with the quality classification of leys and fallows, and it is likely that the classification of the structural crop types used to calculate crop diversity suffers less from such problems. Although such ambiguities should preferably first be resolved, the fact that effects of one action may be less predictable than another in terms of variability in effects among species should weigh in on the decision among alternatives. In our example, although the average effect is weaker, crop diversity may be viewed as a safer alternative as all species are likely to respond similarly, while if aiming for more ley and fallow, species are more variable in their response and it is more likely that it is negative. Second, under the assumption of a normal distribution of effects of land use among species, acquiring estimates of its mean and standard deviation would allow predictions to be made about potential effects also for species not included in the modeling but that have similar ecology as the species included.

### Joint modeling and residual variation and covariation

The joint modeling allowed taking potential interactions among species into account thereby reducing the risk of over‐ or underestimating effects of land use predictors (Clark et al. 2014). Effects for individual species could be underestimated if the relationship between land use and abundance is dampened by competition between species or, conversely, overestimated if species facilitate each other. Whether or not species interact directly, correlations could also result from similar or opposite responses to factors that are not measured or included in the analysis. In our analysis, we did not find support for residual correlations among species. This suggests that the species included do not interact with each other at the scale studied (landscapes of 314 ha) and/or at a level strong enough to influence abundance. Arguably, our power to detect correlations is limited by low counts for several species. On the other hand, low numbers may preclude the potential for individuals to interact. Our results also suggest that there are no other factors affecting farmland bird abundance in similar or opposite ways that we have failed to include. This is not to say that all relevant predictors were included in our model. Rather, many species showed considerable residual variation (σ_*j*_) indicating that these species are idiosyncratically affected by factors that have been left out. There is, hence, great potential to find further characteristics of land use management that could be targeted by interventions.

We note that under certain priors (IW) and distributional assumptions (Poisson), we did find indications of residual correlations among species (Fig. 4a). As judged from differences in WAIC, however, these models were consistently over‐parameterized compared to the best performing model. Arguably, not accounting for residual variation in species counts, as was the case under a Poisson model, might have resulted in that this variation was incorporated as covariance in abundance through the use of an inverse Wishart prior which is known to introduce dependence between variance and covariance (Alvarez et al. 2014, Liu et al. 2016). Specifically modeling extra variation through the negative binomial model in combination with using priors where the variance‐covariance dependence was broken indicated that this was indeed the case and that our data did not show compelling evidence for correlations between species.

## Conclusions

Our results suggest that conservation actions (e.g., from agri‐environmental subsidies or cross‐compliances) aimed specifically at land in production could provide benefits to most farmland birds. Hence, our results could inform the development of future agricultural policies such as CAP to identify measures that could be supported by agri‐environment schemes or be part of cross compliance. Potential actions need, however, to take the landscape context into consideration, suggesting that future policies would benefit from a better spatial targeting of interventions. Actions should be directed toward regions where an increased diversity or reduced intensity gives the largest contrasts, which is generally where availability of more‐natural habitats is scarce. Applying intensity‐reducing actions in already heterogeneous environments may rather have negative effects by reducing habitat resources for species adapted to more or less intensive agriculture.

We found several advantages of modeling species jointly in order to assess impact on biodiversity. For example, modeling of land use dependence as coming from a common distribution aided in estimating effects also for rare species. The borrowing of information seemed in general to make analyses more robust as estimates of land use dependence was very similar across models irrespective of priors or model complexity. It also provided direct information about both the average effect of studied predictors and its variability among species without any need to make post‐analysis summaries. Our results indicated the importance of prior and distributional assumptions, because weak or structured priors (IW) as well as conservative distributions may confound residual variation for correlations overstating the importance of species interactions. In general, we show the ability of joint species modeling to handle a wide array of bird species and account for the distribution of their monitoring data, which makes it well suited for evaluating agricultural policies such as CAP, by linking it to land use models that captures consequences of contra factual policy scenarios.

## Data Availability

Data are available at the Swedish National Data Service: https://doi.org/10.5878/58w0-m352

## Note

1
https://www.ebcc.info/pan-european-common-bird-monitoring-scheme-pecbms/
