# Supplementary material for: Estimating effects of arable land use intensity on farmland birds using joint species modeling
Source: Ecol Appl. 2019 Mar 15;29(4):e01875. doi: 10.1002/eap.1875 (PMC6850359; doi:10.1002/eap.1875)

Supporting Information. Martin Stjernman, Ullrika Sahlin, Ola Olsson, and Henrik G. Smith. 2019. Estimating effects of arable land-use intensity on farmland birds using joint species modeling. *Ecological Applications.*

Appendix S1

Table S1. Description of the classification of crops defined in IACS into structural crop classes used when calculating Shannon crop diversity.

Structural crop class	***IACS crop class***
Spring-sown cereals	Barley (spring)<br>Wheat (spring)<br>Oats<br>Cereal trials<br>Mixed cereals
Autumn-sown cereals	Barley (autumn)<br>Wheat (autumn)<br>Triticale<br>Rye
Spring-sown oil-seeds	Oil-seed rape (spring)<br>Turnip rape (spring)
Autumn-sown oil-seeds	Oil-seed rape (autumn)<br>Turnip rape (autumn)
Maize	Maize
Potatoes	Starch potatoes<br>Ware potatoes
Vegetables	Vegetables
Sugar beets	Sugar beets<br>Fodder beets (mangel beets)
Seed leys	Seed leys (annual)<br>Seed leys (perennial)
Energy crops	Salix<br>Poplar<br>Hybrid aspen
Flowering crops	Buckwheat<br>Oil-seed(fodder) radish<br>Fruits<br>Berries<br>Herbs<br>Sunflower<br>White mustard<br>Broad beans<br>Soybean<br>Strawberries

	Sweet lupin<br>Linseed<br>Flax<br>Peas
Fallow	Fallow
Ley	Rotational ley (approved for subsidy)<br>Non-rotational (non-approved for subsidy)

Figure S1. Comparison of land use effects from a full model and a reduced model without Extensive quality and Proportion spring-sown. Points show means (a) and widths of CI (HPD interval) (b) of posterior samples of effects included in both models (intercepts are not included). MA is major axis regression, cor is Pearson correlation coefficient. Black line is 1:1 line.

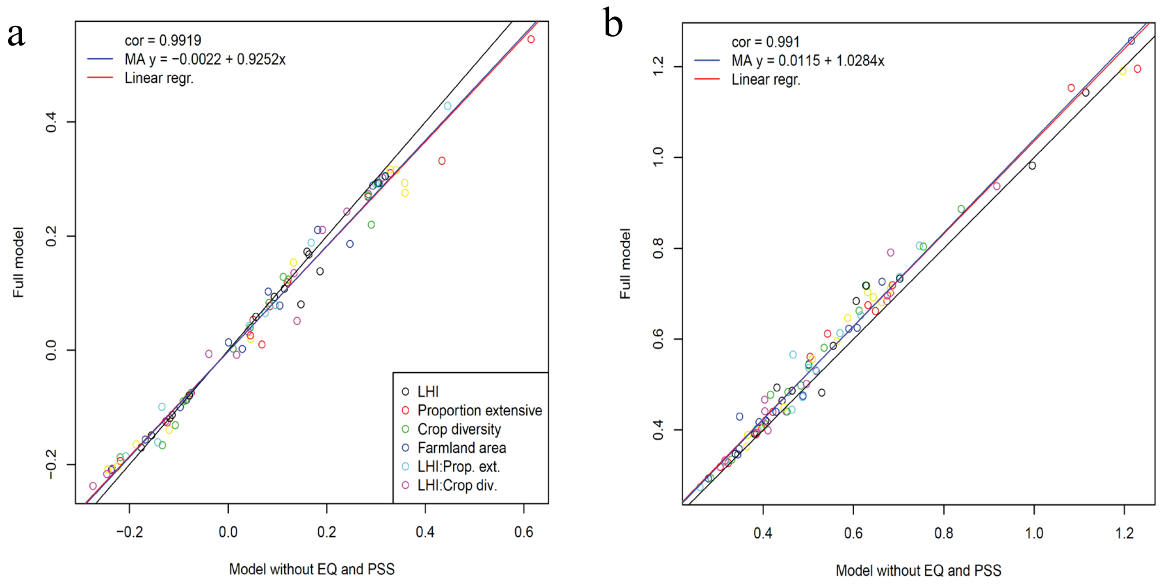

Supplement: Supplementary file 1 [file EAP-29-na-s001.pdf]
